# Florfenicol and oxazolidone resistance status in livestock farms revealed by short- and long-read metagenomic sequencing

**DOI:** 10.3389/fmicb.2022.1018901

**Published:** 2022-10-20

**Authors:** Xue Yang, Tiejun Zhang, Chang-Wei Lei, Qin Wang, Zheren Huang, Xuan Chen, Hong-Ning Wang

**Affiliations:** Animal Disease Prevention and Food Safety Key Laboratory of Sichuan Province, College of Life Sciences, Sichuan University, Chengdu, China

**Keywords:** nanopore sequencing, Illumina sequencing, florfenicol, oxazolidinone, genetic context

## Abstract

Antibiotic resistance genes (ARGs) as a novel type of environmental pollutant pose a health risk to humans. Oxazolidinones are one of the most important antibiotics for the treatment of Gram-positive bacterial infections in humans. Although oxazolidinones are not utilized in the livestock industry, florfenicol is commonly used on farms to treat bacterial infections, which may contribute to the spread of the *cfr*, *optrA*, and *poxtA* genes on farms. Using metagenomics sequencing, we looked into the antibiotic resistome context of florfenicol and oxazolidinone in 10 large-scale commercial farms in China. We identified 490 different resistance genes and 1,515 bacterial genera in the fecal samples obtained from 10 farms. Florfenicol-resistant *Kurthia*, *Escherichia*, and *Proteus* were widely present in these samples. The situation of florfenicol and oxazolidone resistance in pig farms is even more severe. The total number of genes and the abundance of drug resistance genes were higher in pigs than in chickens, including *optrA* and *poxtA*. All the samples we collected had a high abundance of *fexA* and *floR*. Through nanopore metagenomic analysis of the genetic environment, we found that plasmids, integrative and conjugative element (ICE), and transposons (Tn*7*-like and Tn*558*) may play an important role in the spread of *floR*, *cfr*, and *optrA*. Our findings suggest that florfenicol and oxazolidinone resistance genes have diverse genetic environments and are at risk of co-transmission with, for example, tetracycline and aminoglycoside resistance genes. The spread of florfenicol- and oxazolidinone–resistant bacteria on animal farms should be continuously monitored.

## Introduction

Antibiotics have been used extensively for more than half a century as effective medicines for the treatment and prevention of human and animal diseases, as well as to accelerate animal growth. However, with the extensive use of antibiotics, the global situation of drug resistance became progressively worse (Zhang et al., 2015; He et al., 2019). It is estimated that antimicrobial resistance will kill 10 million people per year by 2050, according to O’Neill (2014).

Florfenicol is a synthetic animal-specific drug that has been approved by the Chinese government in 1999. However, due to improper use, *Escherichia coli* resistant to florfenicol started to emerge, and the prevalence of drug-resistant strains increased year after year, garnering a lot of attention. Several florfenicol resistance genes have been found, including *floR*, *fexA*, *fexB*, *cfr*, and others (Arcangioli et al., 1999; Liu et al., 2012; Shen et al., 2013). Linezolid is a first-generation oxazolidinone antibiotic with a potent antibacterial action against Gram-positive infections, commonly known as the last line of defense against infections by multidrug-resistant Gram-positive pathogens (Bozdogan and Appelbaum, 2004). In 2006, the *cfr* gene was found to mediate linezolid resistance (Long et al., 2006). New resistance genes (*optrA* and *poxtA*) can be resistant to oxazolidinones and amide alcohols (Wang et al., 2015; Antonelli et al., 2018). Animal usage of linezolid has never been authorized. Interestingly, three linezolid resistance genes (*cfr*, *optrA*, and *poxtA*) were detected in bacteria of animal origin (Shen et al., 2013; Wang et al., 2015; Lei et al., 2019). It is unclear whether the use of florfenicol has led to the emergence of linezolid-resistant genes. Serious public health concerns have been raised as a result of the widespread distribution of linezolid resistance genes in bacteria.

Continuous monitoring of drug resistance requires not only identifying the microbial resistance group and the host of the resistant group, but also the evaluation of mobile genetic elements (MGEs) (Frost et al., 2005). Pure culture isolation in combination with whole-genome sequencing has been and continues to be a major tool for determining phenotypic and genotypic correlations in the pathogenic bacteria and identifying MGEs with which they are related (Zhao S. et al., 2016; Guo et al., 2017; Xia et al., 2017). However, only a small percentage of bacteria in feces can be cultured and isolated, and this proportion does not reflect the general state of the sample, severely limiting the use of pure culture isolation to investigate the fecal resistome (Luo et al., 2017).

The diversity and abundance of ARGs in samples have considerably risen, thanks to metagenome sequencing (Ma et al., 2016). Although Illumina sequencing has a high level of precision, the read length is short, making it challenging to determine the species and genetic background of ARG. In comparison to short-read sequencing, Oxford Nanopore Method’s (ONT) long-read sequencing technology has a significantly high sequencing error rate, but can generate long reads that can span most repetitive sequences and link the ARGs and their flanking regions, and thus, the knowledge and technology gap mentioned above can be bridged (Che et al., 2019; Li et al., 2022). This study utilized Nanopore and Illumina sequencing technology to evaluate the genomic location of florfenicol ARGs more efficiently and correctly.

## Materials and methods

### Sample collection and pretreatment

Five swine fecal samples and five chicken fecal samples were collected separately from 10 industrialized farms. Chicken samples were collected from four provinces, including Sichuan, Chongqing, Anhui, and Beijing. All the swine farms were located in Sichuan province (Supplementary Table 1). We selected five houses on each farm and used the five-point sampling method to collect the samples in each house and mix them into one. Samples were moved from the field to the lab in a low-temperature box with dry ice. Five samples from each farm were combined into one. Five grams of mixed samples from each farm was weighed, mixed thoroughly by adding 5 ml of phosphate-buffered saline (PBS), and filtered through gauze.

### Cultivation of resistant bacteria, DNA extraction, and detection

Approximately, 100 μ L of the filtrate was plated onto an Luria-Bertani (LB) agar plate supplemented with 16 μ g/mL florfenicol. After incubating overnight (12 h) in aerobic conditions at 37°C, each sample was collected by washing all colonies/isolates grown on the incubated LB plate three times using PBS. DNA was extracted from the 10 post-culture sample colonies collected above using the QIAampPower fecal DNA kit (QIAGEN, Netherlands)^1^ according to the manufacturer’s instructions. These DNA samples were extracted for subsequent metagenomic sequencing. For library building and sequencing, sufficient supplies of high-quality nucleic acids must be obtained. We used agarose gel electrophoresis (AGE) to assess the DNA purity and integrity, and Qubit to determine the DNA concentration.

### Library preparation, sequencing, and assembly

#### Illumina library preparation and sequencing

For the DNA sample preparation, a total of 1 μg of DNA per sample was used as the input material. Sequencing libraries were created using the New England Biolabs (NEBNext)^®^ UltraTM DNA Library Prep Kit for Illumina (NEB, USA). After passing the library check, Illumina sequencing using the PE150 strategy was performed at Novogene Corporation (Beijing, China).

#### Illumina data pretreatment and metagenome assembly

To obtain the clean data for further analysis, Readfq (V8^2^) was used to pre-process the raw data. Clean data must be blasted to the host database, which uses the Bowtie 2.2.4 software^3^ with default parameters to filter reads from the host and remove the reads with high alignment similarity. MEGAHIT^4^ was used to assemble clean data created from each sample from scratch.

#### Nanopore library preparation and sequencing

According to the Illumina sequencing results, two samples (C.2 and S.4) with a high abundance of drug resistance genes were chosen for nanopore sequencing.

For the MinION platform (Oxford Nanopore Technologies, Oxford, UK), using a total of 8 μg of DNA per sample as input material, sequencing libraries were created using the Ligation Sequencing Kit (SQK-LSK109). The DNA was disrupted with a Megaruptor (Diagenode, Denville, NJ, USA), and the fragments larger than 10 kb were screened with Bluepippin. The barcodes were applied after the end repair and addition of the A tail, and then the fragment length was detected. The linker and template were then purified, and samples with different barcodes were combined in equimolar quantities. The clustering of the bar-coded samples was performed with PromethION Flow Cell Priming Kit (EXPFLP001.PRO.6) according to the manufacturer’s instructions.

#### MinION data pretreatment and metagenome assembly

The original format of nanopore sequencing down data is fast5 format, and fast5 was converted to fastq format using Guppy (Oxford Nanopore built-in software) basecalling. We used the software NanoPlot (Version: NanoPlot 1.18.2) selection threshold *Q* > 7 for quality control of the data. The blasr (version 5.3.5^5^) software was used to remove host contamination by default.

Flye (Version: 2.4.2-release^6^) was used to assemble and analyze the clean data. Following data preprocessing, the assembled scaftigs were compared to the clean data of each sample by Bowtie 2.2.4 (see text footnote 2) to obtain the paired-end reads (PE) reads that were not used. Filter scaftig fragments that are less than 500 bp in length for statistical analysis resulting from a single assembly.

### Bioinformatics analysis

#### Gene prediction and abundance analysis

The open-reading frame (ORF) predictions for all assembled scaftigs (>500 bp) were performed using MetaGeneMark (V2.10^7^) and filtered for length information less than 100 nt. The predicted ORFs were de-redundant using the cd-hit program in the CD-HIT software (V4.5.8^8^). These procedures are used to produce a non-redundant gene catalog (Unigenes).

#### Species and resistance gene annotation

Species annotation: DIAMOND (V0.9.9^9^) compares Unigenes with bacteria, fungi, and archaea extracted from National Center for Biotechnology Information (NCBI’s) NCBI RefSeq (NR) (Version: 2018.01) database. Species annotation information was determined to use the systematic classification function of the MEGAN6.^10^

Resistance gene annotation: Unigenes were aligned with the Comprehensive Antibiotic Resistance Database (CARD) using resistance gene identifier (RGI) (v4.2.2) to identify antibiotic resistance genes (ARGs). The relative abundance of each ARO was computed using the RGI alignment findings and the abundance data from Unigenes. All analyses make use of the default settings.

#### Other analysis

We perform principal component analyze (PCoA) analysis based on Bray--Curtis distance. The distribution patterns of the bacterial genera of samples were also clustered and characterized using a principal component analyses (PCoAs) approach by R package and QIIME.^11^ The Pearson correlation test was performed using the Hmisc software package of R language to analyze the correlation between ARGs. The correlation was screened using the threshold of *P* < 0.01, *r* ≥ 0.9, and then visualized using Gephi 0.9.2.^12^ The histogram is drawn using the ggplot2 package in R software, and the circle chart is drawn using the circos.^13^ MinION-assembled contigs were searched for MGEs against CARD database, and the location of the contig on the chromosome or plasmid was ascertained using plasflow.^14^ The genetic environments were analyzed using rast website,^15^ Easyfig v2.2.2,^16^ and Adobe Illustrator 2020 software.

## Results

### Illumina and MinION sequencing read statistics

Illumina sequencing on average generated 12.28 GB of clean data with an average of 74,815 scaftig numbers (Supplementary Table 2). For the two samples (C.2 and S.4) for which the nanopore data assembly was performed, the length of the nanopore reads (avg. 52 kb; N50 198 kb) was much longer than the contigs (avg. 1.3 kb; N50 1.8 kb) assembled using Illumina short reads (Supplementary Table 3). A total of 11 long scaftigs over 2 GB were generated, and these sequences may represent the complete genome of the strain. With the use of these longer nanopore readings, we can examine the genomic localization of ARGs in more completely assembled sequences.

### Composition of fecal communities

**FIGURE 1:**
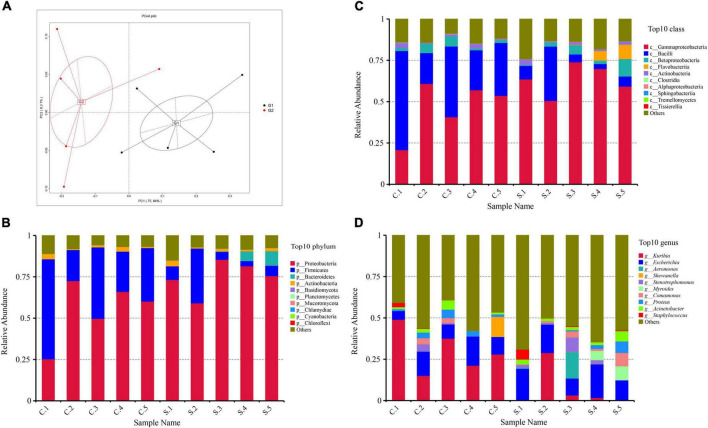
**(A)** Principal component analyze (PCoA) of the 10 samples based on Bray–Curtis distance. **(B)** Relative abundance of the top 20 phyla; **(C)** Relative abundance of the top 20 classes; **(D)** Relative abundance of the top 20 genera.

The PCoA intuitively displayed the differences in microbial communities between different samples and two groups (G1 indicates the chicken farms and G2 indicates the swine farms). The microbial communities from G1 and G2 have a big difference (Figure 1A), and the number of genes in swine farms is higher than that in chicken farms.

At the phylum rank, the compositions of the florfenicol-resistant microbial communities in each sample were relatively similar. Proteobacteria (65%), Firmicutes (23%), and Bacteroidetes (15%) were the most abundant bacterial phyla in all 10 samples (Figure 1B). The predominant classes were Gammaproteobacteria (55%), Bacilli (23%), and Betaproteobacteria (4%) (Figure 1C). The predominant genera were *Kurthia* (19%), *Escherichia* (14%), and *Proteus* (2.4%) (Figure 1D). At the genus level, the drug-resistant bacteria in the feces of pigs and chickens are quite different. The main resistant bacteria in the chicken farms is *Kurthia*, while in the pig farms it is *Escherichia*. Florfenicol-resistant *Staphylococcus* is more abundant in pig farms than that in chicken farms. Some samples also displayed unique features, such as a high abundance of *Staphylococcus* in S1 and an unusually high abundance of *Aeromonas* and *Stenotrophomonas* in S3 (Supplementary File 1).

### Composition of the resistome

**FIGURE 2:**
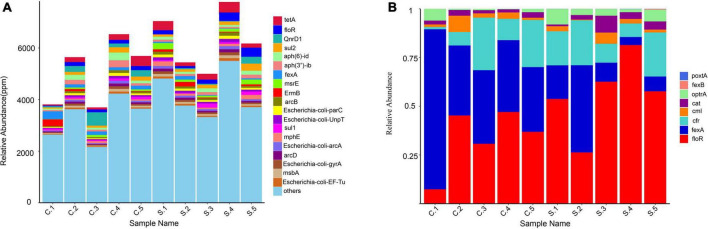
**(A)** Relative abundance of the top 20 antibiotic resistance genes (ARGs); **(B)** Relative abundance of resistance genes associated with florfenicol.

A total of 490 different resistance genes were detected in 10 samples; *tet*(A), *qnrD*, *floR*, *sul2*, *erm*(C), and *tetM* were the predominant resistance types in all the samples (Supplementary File 2). The abundance of total drug resistance genes and the abundance of *floR* in pig farms were significantly higher than in chicken farms. Chicken farms have a higher abundance of *fexA* (Figure 2A).

We estimated the relative abundance of associated genes for the clear analysis of bacterial resistance to florfenicol and oxazolidinone in pig and chicken farms. *FloR, fexA*, and oxazolidinone resistance genes *cfr* and *optrA* are distributed on all farms. *PoxtA* was distributed in nine farms except for C3, although its abundance was low. The abundance of *optrA* and *poxtA* in swine farms was higher than that in chicken farms. The prevalence of chloramphenicol resistance genes *cml* and *cat* remained relatively high (Figure 2B).

**FIGURE 3:**
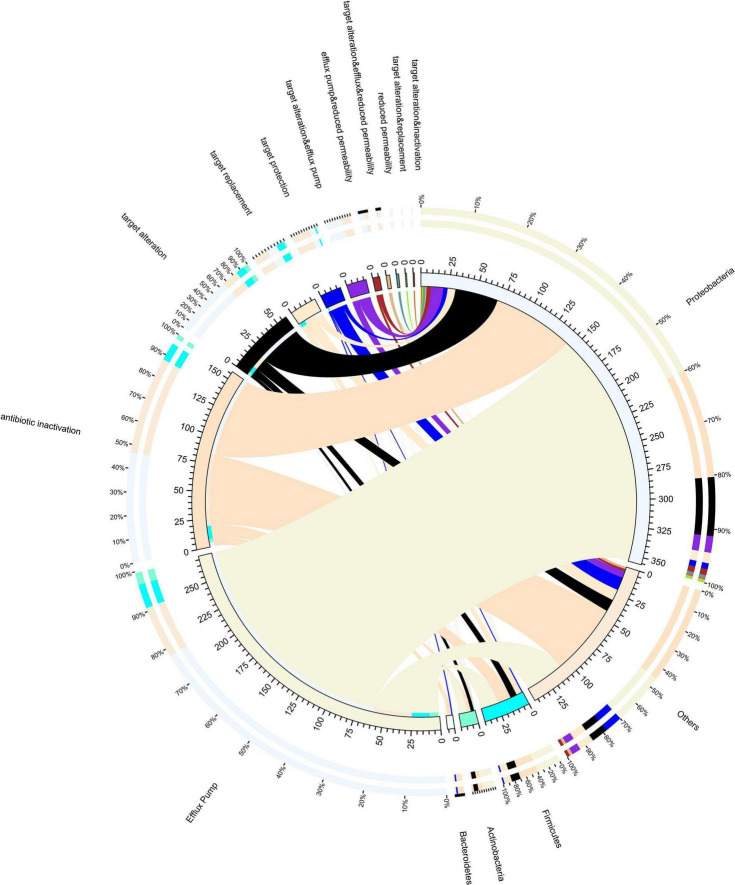
Resistance mechanisms and species circle map. On the right is the phylum-level species information, and on the left is the resistance mechanism information. Different colors of the inner circle indicate different species and resistance mechanisms of resistance, and the scale is the number of genes.

In this study, the resistance mechanism results were based on CARD databases (Figure 3). In our study, resistance mechanisms were classified into five major categories, namely, efflux pump, antibiotic inactivation, target alteration, antibiotic target protection, and antibiotic target replacement. Additional combinatorial mechanisms also exist. According to our results, antibiotic efflux pumps were detected as the main resistance mechanism in all samples, followed by antibiotic inactivation and target alteration. Proteobacteria have the most resistance genes, and the resistance mechanism is mainly by efflux pumps. It is noteworthy that many chromosomal genes are included in the CARD database. It is problematic as to whether chromosomal genes should be counted as ARGs, since they are resistant only when a mutation occurs, for example, quinolone resistance genes (*gyrA* and *parC*).

**FIGURE 4:**
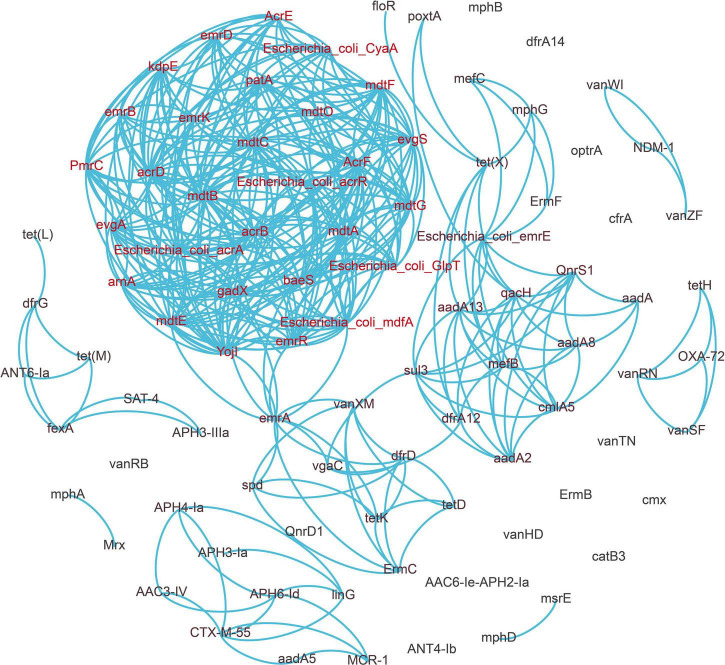
Network analysis revealing the co-occurrence patterns between antibiotic resistance genes (ARGs). A connection represents a strong relationship between ARGs (Pearson’s correlation coefficient *r* ≥ 0.9, *p* < 0.01).

We selected high abundance resistance genes and clinically important resistance genes to construct the network map (Figure 4). We found that a co-occurrence relationship existed among many drug resistance genes. It is particularly noteworthy that the florfenicol resistance gene *floR* has a strong correlation with the tetracycline resistance gene *tet*(X), and *fexA* is also positively correlated with *tet*(M) and aminoglycoside resistance gene *aph3-llla*. *PoxtA*, a crucial oxazolidinone resistance gene that has undergone extensive research in recent years, has a favorable correlation with both *tet*(X) and *qacH*.

### Genetic environment of *floR*, *cfr*, and *optrA*

A total of 506 drug resistance genes were detected in the two samples (C.2 and S.4) revealed by nanopore sequencing. We discovered that ARGs on plasmids made up 32% of all ARGs using the software plasflow, indicating that they have a high potential for transfer into recipient settings. To systematically observe the resistance genes and genetic environment of florfenicol and oxazolidinone, we use nanopore sequencing reads to analyze relevant resistance genes located on MGEs. Specific information on the positioning of *floR*, *cfr*, *optrA*, *and fexA* is shown in Supplementary File 3. The genetic analysis of the selected 12 reads showed that these genes were associated with plasmids, integrative and conjugative element (ICE), and transposons.

**FIGURE 5:**
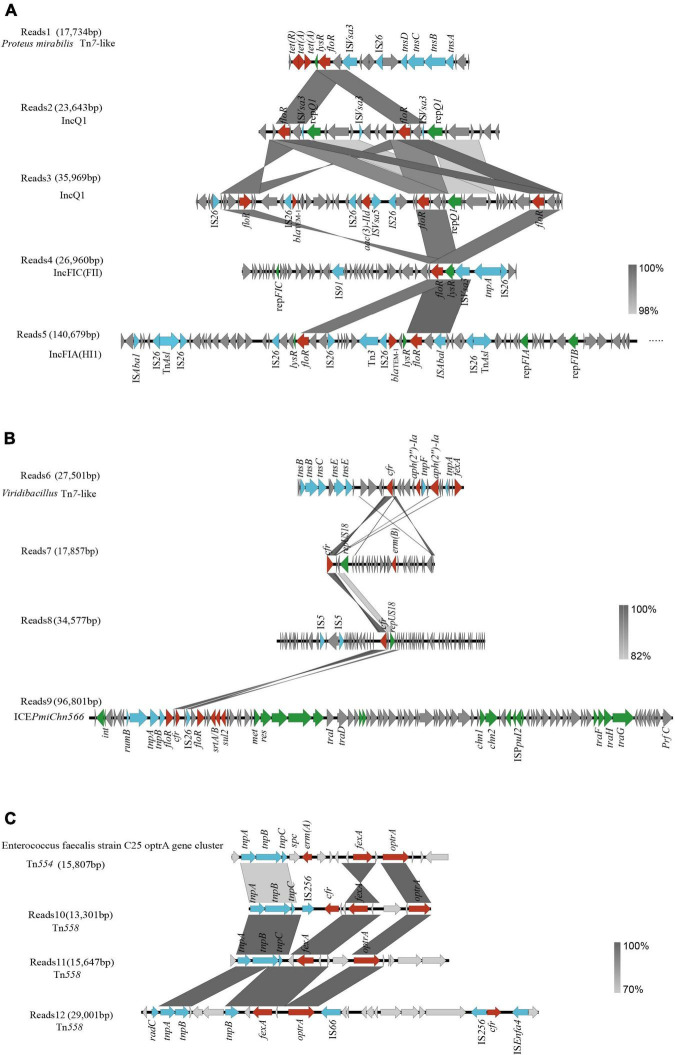
Genetic context of *floR*, *cfr*, and *optrA* genes. **(A)** *floR* genetic context; **(B)** *cfr* genetic context; **(C)** *optrA* genetic context. Genes and open-reading frames (ORFs) are shown as arrows, and their orientations of transcription are indicated by the arrowheads. Antimicrobial resistance genes are in red, transposase elements are in blue, replication genes and other protein genes are in green, and hypothetical protein genes are in gray.

Four different chromosomal and plasmid-harbored *floR* clusters were found. First, we found that *floR* is located in Tn*7*-like transposons from *Proteus mirabilis* in Reads1. Reads2–Reads5 represent three plasmids carrying *floR* (Figure 5A). They harbored the replication initiator genes *repQ1*, *repFIC*, *repFIA*, and *repFIB*, respectively. On the majority of plasmids, we could locate more than one *floR* gene. Other resistance genes, including *bla*_TEM–1_, *aac(3)-IId*, *tet*(A), and *tet*(R), were also found in these genomes. We discovered that IS*26* and IS*Vsa3* were frequently present in the surroundings, indicating that IS*26* and ISV*sa3* might promote the transfer of *floR* among different plasmids or other MGEs.

Reads6 harbored a *cfr*-carrying gene fragment (Figure 5B). The BLAST analysis showed that it was 99.81% identical to the corresponding region of *Macrococcus caseolyticus* strain 5459_5_49 (GenBank accession no. BK012114) with 48% coverage. The transposase gene tn*sABCDE* is the intrinsic structure of the transposon Tn*7* (Luo et al., 2021). The chromosomal location and the presence of tn*sABCDE* suggest that *cfr-aph(2″)-Ia-fexA* is carried in *Viridibacillus* by a novel transposon distantly related to Tn*7*.

Two *cfr*-carrying plasmids with sizes ranging from 17.8 to 34.5 kb were obtained after assembly (Reads7 and Reads8). They carried the replicon *repUS18* (Figure 5B). A previous report showed that the *repUS18* gene has been linked to the antimicrobial resistance gene transfer in *Enterococci*, and is frequently found on broad host-range Inc18 plasmids (Luo et al., 2021). We also found a novel complete SXT/R391 ICE in the genome of *P. mirabilis* named ICE*PmiChn566* (Figure 5B; He et al., 2021a). ICE*PmiChn566* is 96,801 bp in size, with a G+C content of 47.5%. BLAST analysis showed that ICE*PmiChn566* had 99.94% nucleotide identity to ICE*PmiChn1* with 96% coverage (Lei et al., 2016). ICE*PmiChn566* carries *cfr* and lacks *tet*(G)*-tetR* region compared with ICE*PmiChn1*.

The horizontal transfer of the *optrA* is mostly influenced by the genetic environment. In addition to IS*1216*, which has a significant impact on the transmission of *optrA*, the genetic environment surrounding *optrA* might include Tn*558* or Tn*554* which consist of three transposase-encoding genes (*tnpA*, *tnpB*, and *tnpC*) (He et al., 2016). Our research also confirms this fact, that is, Reads11 and 12 (Figure 5C). Interestingly, we found (Reads10) a Tn*558* connected downstream to *cfr-fexA-optrA* (Figure 5C). As far as we know, the localization of *cfr-fexA-optrA* on a single Tn*558* was first reported in China. BLAST analysis showed that Reads10 was 99.82% identical to the corresponding region of *Enterococcus faecium* strain C25 *optrA* gene clusterl sequence (GenBank accession no. MK251150) with 32% coverage (Kang et al., 2019).

## Discussion

Antimicrobial resistance varies dramatically across countries, regions, and even individual herds (Zhou et al., 2022). Researchers in Hong Kong made significant progress in their investigation of the antibiotic resistome in four wastewater treatment plants using Nanopore and Illumina sequencing techniques (Che et al., 2019). We adopted their methods to investigate the composition, and abundance of fecal microbial and resistome composition in chickens and pigs from large-scale breeding farms in China. The findings of this study add to our understanding of the breadth and extent of florfenicol and oxazolidinone resistance in China’s large-scale industrial swine and chicken farms.

We were able to identify and quantify a total of 60 phyla, 72 classes, and 1,515 bacterial genera that can exist under the selection pressure of florfenicol in all of the samples using a metagenomics sequencing technique. The population of Bacteroidia was the most numerous in uncultured stool samples (Munk et al., 2018). But Proteobacteria has risen to the top of the food chain in our samples. It should not be overlooked that our experiments were carried out under aerobic conditions, which are highly selective for species abundance. The low abundance of Bacteroidetes in the samples should be attributed to the aerobic culture conditions used in this study, as Bacteroidetes are anaerobic. In contrast, the widespread presence of *Kurthia*, *Escherichia*, and *Proteus* in these samples is not only due to their high resistance to florfenicol but is also related to aerobic culture. The oxazolidinone antibiotic linezolid has effective antibacterial activity against *staphylococci*. The presence of a high abundance of florfenicol-resistant *staphylococci* in animal feces is a phenomenon that deserves our continued attention.

In our samples, compared to chicken farms, swine farms have a higher total number of genes and gene abundance for resistance to florfenicol and oxazolidinone, and there are significant variances between farms. On the one hand, it is related to the differences in the structure and composition of the intestine between pigs and chickens. On the other hand, this may be attributed to the huge use of florfenicol in pig farms and the prohibition of the use of antibiotics in laying hens during the laying period (Zhao et al., 2010; Zhao Q. et al., 2016; Munk et al., 2018). Previous research has shown although the use of chloramphenicol in food-producing animals was banned in China in 2002,^17^ florfenicol is still in use, which may cause a still relatively high prevalence of the chloramphenicol resistance genes (Yang et al., 2022). The *cml* and *cat* genes were also found to be highly abundant in our study. In this study, the main resistance mechanism of these resistant bacteria was found to be the efflux pump. Through correlation analysis, we discovered extensive colinearity of florfenicol and oxazolidinone resistance genes with other resistance genes, such as tigecycline and aminoglycoside.

Importantly, long sequences make it possible to analyze the genetic context of the multiple drug resistance genes in binned genomes. At the same time, we recognize that ARGs localized on MGEs may be at risk of transmission. Our study demonstrates that plasmids, ISs, transposons, and ICE all contribute significantly to facilitating the dissemination of *floR*, *cfr*, and *optrA* genes. The genetic structures of the drug-resistant plasmids were different, indicating the diversity of plasmids carrying the *floR* and *cfr* genes. IncF and IncQ plasmids are among the most prevalent incompatibility types involved in the transfer of resistance determinants in Enterobacteriaceae and have been reported worldwide (Muthuirulandi Sethuvel et al., 2019). LncQ and lncF carriage of *floR* has also been widely identified and reported, which is also compatible with our findings (Loftie-Eaton and Rawlings, 2012; Mohsin et al., 2021).

By comparison, it was found that the tetracycline resistance genes [*tet*(A) and *tet*(R)] and the beta-lactam antibiotic resistance gene *bla_TEM–1_* frequently existed on the plasmid containing the *floR* gene, indicating that there may be co-transmission between these resistance genes. At the same time, it was also found that multiple insertion sequences IS*26* and IS*Vsa3* appeared on the plasmid carrying the *floR* gene, which may spread through plasmid recombination and mediate bacterial multidrug resistance. One study found the existence of IS*Vsa3* on the reported ICE carrying the *floR* gene and verified that IS*Vsa3* mediates the transfer of *floR* (He et al., 2021b). The mobile element IS*26*, a member of the IS*6* family, is essential for the recombination, clustering, and spread of resistance genes (Liu et al., 2021). ICE*PmiChn566*, which had 99.94% nucleotide identity to ICE*PmiChn1*, has one more *cfr* gene added to VRn (He et al., 2015; Liu et al., 2021). Most likely, the IS*26* insertion sequence mediated the transfer of this *cfr* region within the chromosome. *FloR* and *cfr* were carried in the Tn*7*-like structure, which indicated that Tn*7*-like elements might play a role in mediating *floR* and *cfr* transmission. Tn*558* has been an important dissemination media of *optrA* and has been reported all over the world (Tsilipounidaki et al., 2019; Almeida et al., 2020a,b). Tn*558* normally surrounds *optrA* and mediates their transfer (He et al., 2016). In our study, we identified a genetic environment downstream of Tn*558* as *cfr-fexA-optrA.* We should take appropriate measures to control the further spread of *cfr* and *optrA* and the emergence of mutant linezolid resistance.

These findings reveal that florfenicol and oxazolidone resistance is a serious issue in pig and chicken farms. Diverse resistant bacteria and resistant genes exist in farms, and these drug-resistant genes have a high risk of transmission. Antibiotic resistance in farms could pose a significant hazard to the environment, food safety, and human health. We should conduct continuous monitoring of drug-resistant bacteria, drug-resistant genes, and MGEs on farms. In addition, adequate management of animal waste in farms should be implemented, and florfenicol usage should be limited to prevent the spread of antibiotic resistance.

## Conclusion

In this study, we applied Illumina and Nanopore metagenomic sequencing techniques to investigate the ARGs and genetic contexts of ARGs related to florfenicol and oxazolidinone across 10 farms in China. The findings revealed a wide spread of florfenicol-resistant bacteria and ARGs in feces, including *floR*, *fexA*, and the oxazolidinone resistance determinants *cfr*, *optrA*, and *poxtA*. More substantial drug resistance issues exist in pig farms than in chicken farms. MGEs, including plasmids, ICE, transposons, and insertion sequences, all play a role in the dissemination of these genes, according to genome binning and genetic context analysis. Our results demonstrate that the florfenicol and oxazolidone resistance genes are closely associated with other important resistance genes, such as tigecyclines and aminoglycoside antibiotics. Pig and chicken farms are an important repository of oxazolidinone resistance genes.

## Data availability statement

The datasets presented in this study can be found in online repositories. The names of the repository/repositories and accession number(s) can be found below: https://www.ncbi.nlm.nih.gov/search/all/?term=PRJNA843911 and https://www.ncbi.nlm.nih.gov/search/all/?term=PRJNA843901.

## Author contributions

XY and C-WL conceived the study, designed the experiments, and analyzed the data. TZ analyzed the data. QW, ZH, and XC performed the experiments. XY wrote the manuscript. H-NW guided the experiments and revised the manuscript. All authors contributed to the article and approved the submitted version.

## Footnotes

1
https://www.qiagen.com/au/products/new-products/qiaamp-powerfecal-pro-dna-kit/

2
https://github.com/cjfields/readfq

3
http://bowtie-bio.sourceforge.net/bowtie2/index.shtml

4
https://github.com/voutcn/megahit

5
https://anaconda.org/bioconda/blasr

6
https://github.com/fenderglass/Flye/

7
http://topaz.gatech.edu/GeneMark/

8
http://www.bioinformatics.org/cd-hit

9
https://github.com/bbuchfink/diamond/

10
https://software-ab.informatik.uni-tuebingen.de/download/megan6/welcome.html

11
https://qiime2.org/

12
https://gephi.org/

13
http://circos.ca/software/download/

14
https://plasflow.co.uk/

15
https://rast.nmpdr.org/

16
http://mjsull.github.io/Easyfig/

17
http://www.moa.gov.cn/ztzl/ncpzxzz/flfg/200709/t20070919_893091.htm

## Supplementary material

Supplementary Table 1 Sample information.

Supplementary Table 2 Illumina sequencing supplementary data.

Supplementary Table 3 Nanopore sequencing supplementary data.

Supplementary File 1 Species information (Illumina sequencing).

Supplementary File 2 ARGs relative abundance (Illumina sequencing).

Supplementary File 3 MGE information of florfenicol resistance gene mapping.
