# Supplementary material for: Florfenicol and oxazolidone resistance status in livestock farms revealed by short- and long-read metagenomic sequencing
Source: Front Microbiol. 2022 Oct 20;13:1018901. doi: 10.3389/fmicb.2022.1018901 (PMC9632178; doi:10.3389/fmicb.2022.1018901)
Supplement: Supplementary Table 1 — Sample information. [file Data_Sheet_1.zip › Supplementary Tables 1 -3.DOCX]

Supplementary file 1

TableS1. Sample information

| **sample name** | **source** | **group** | **Collection location** | **antimicrobial usage background** |
| --- | --- | --- | --- | --- |
| C.1 | fowl | G1 | Sichuan province | enrofloxacin |
| C.2 |  |  | Anhui province | cephalosporin  enrofloxacin  florfenicol |
| C.3 |  |  | Anhui province | tiamulin  enrofloxacin |
| C.4 |  |  | Chongqing city | spectinomycin  lincomycin |
| C.5 |  |  | Beijing city | unknown |
| S.1 | swine | G2 | Sichuan province | amoxicillin  tilmicosin |
| S.2 |  |  |  | doxycycline hydrochloride  tilmicosin  florfenicol |
| S.3 |  |  |  | amoxicillin  tilmicosin  florfenicol |
| S.4 |  |  |  | enrofloxacin  gentamicin  florfenicol |
| S.5 |  |  |  | amoxicillin  florfenicol |

| **SampleID** | **RawData**（**G**） | **CleanData**（**G**） | **Total len.(bp)** | **Scaftigs Num.** | **Average len.(bp)** | **N50 Len.(bp)** | **N90 Len.(bp)** | **Max len.(bp)** |
| --- | --- | --- | --- | --- | --- | --- | --- | --- |
| C.1 | 13.68 | 13.67 | 72,054,665.00 | 43,171.00 | 1,669.05 | 2,474.00 | 650.00 | 365,315.00 |
| C.2 | 12.15 | 12.14 | 119,862,902.00 | 90,990.00 | 1,317.32 | 1,507.00 | 604.00 | 174,148.00 |
| C.3 | 11.93 | 11.92 | 83,794,602.00 | 58,572.00 | 1,430.63 | 1,856.00 | 623.00 | 145,667.00 |
| C.4 | 11.82 | 11.81 | 55,954,384.00 | 43,333.00 | 1,291.26 | 1,564.00 | 599.00 | 67,954.00 |
| C.5 | 11.67 | 11.67 | 94,977,650.00 | 59,688.00 | 1,591.24 | 2,309.00 | 645.00 | 262,815.00 |
| S.1 | 12.50 | 12.50 | 92,762,359.00 | 77,393.00 | 1,198.59 | 1,360.00 | 591.00 | 132,496.00 |
| S.2 | 11.99 | 11.98 | 63,874,799.00 | 52,059.00 | 1,226.97 | 1,387.00 | 594.00 | 143,598.00 |
| S.3 | 11.57 | 11.55 | 175,471,439.00 | 135,206.00 | 1,297.81 | 1,535.00 | 598.00 | 109,751.00 |
| S.4 | 12.81 | 12.81 | 120,095,324.00 | 82,235.00 | 1,460.39 | 1,905.00 | 624.00 | 119,321.00 |
| S.5 | 12.81 | 12.79 | 146,649,290.00 | 105,507.00 | 1,389.95 | 1,745.00 | 610.00 | 217,954.00 |

TableS2. Illumina sequencing supplementary data

| **SampleID** | **RawData(G)** | **CleanData(G)** | **Total len.(bp)** | **Scaftigs Num.** | **Average len.(bp)** | **N50 Len.(bp)** | **N90 Len.(bp)** | **Max len.(bp)** |
| --- | --- | --- | --- | --- | --- | --- | --- | --- |
| C.2 | 13.52 | 13.50 | 171,176,086.00 | 3,005.00 | 56,963.75 | 272,694.00 | 28,730.00 | 3,443,830.00 |
| S.4 | 15.14 | 15.13 | 147,168,424.00 | 2,982.00 | 49,352.25 | 133,523.00 | 26,179.00 | 4,219,096.00 |

TableS3. Nanopore sequencing supplementary
